# The tell-tale hearts: Donor-derived invasive fungal infections among orthotopic heart transplant recipients

**DOI:** 10.1016/j.mmcr.2025.100702

**Published:** 2025-03-31

**Authors:** Jay Krishnan, Manuela Carugati, Rachel A. Miller, Cameron R. Wolfe, John R. Perfect, Julia A. Messina

**Affiliations:** Duke University, Division of Infectious Diseases, Department of Medicine, Duke University Medical Center, Durham, NC, USA

**Keywords:** Donor-derived fungal infection, Orthotopic heart transplantation, *Candida parapsilosis* complex, *Aspergillus fumigatus*, Fungal endocarditis

## Abstract

Donor-derived invasive fungal infections among solid organ transplant recipients are rare but sometimes devastating events associated with notable morbidity and mortality. Here we describe two donor-derived fungal infections – one *Candida parapsilosis* complex infection and one *Aspergillus fumigatus* infection – that occurred among heart transplant recipients at a quaternary care center. Both recipients survived their infections, though with substantial morbidity despite aggressive surgical intervention and antifungal therapy.

## 1 Introduction

Donor-derived invasive fungal infections (IFIs) among solid organ transplant recipients are rare occurrences that carry significant morbidity and mortality. A 2020 report from the Organ Procurement and Transplantation Network estimates that unexpected donor-derived fungal infections affected around 2–3 out of every 10,000 solid organ transplant recipients between 2012 and 2017 in the United States [1].

Transmission of both yeasts and molds from donor to recipient has been observed. For instance, between 2012 and 2017, donor-derived *Candida* infections comprised 25 % of the fungal pathogens transmitted via solid-organ transplantation [1]. Contamination of preservation fluid is considered to be the most common cause of donor-derived candidiasis while infection transmission from donors with candidemia is considered rare [2].

Donor-derived *Aspergillus* infections are rarer events. Out of 2185 reports of possible donor transmission of disease, only 3 cases of confirmed donor-to-recipient transmission of *Aspergillus* species occurred between 2012 and 2017 [1]. Case reports of donor-derived *Aspergillus* IFIs among transplant recipients are correspondingly rare. One systematic review from 2013 identified only 24 cases of donor-derived IFI with filamentous fungi [3]. *Aspergillus* species were responsible for over 70 % of these cases, but the majority (>90 %) in this series occurred among renal transplant recipients. To our knowledge, only one additional case of confirmed donor-derived *Aspergillus* IFI in a heart transplant recipient has been reported since 2012 [4].

Here we present two cases of confirmed donor-derived IFI among two heart transplant recipients.

## 2 Case presentations

### 2.1 Donor-derived *Candida parapsilosis* complex infection

#### 2.1.1 Patient presentation

A 54-year-old man status post remote orthotopic heart transplantation for non-ischemic cardiomyopathy complicated by cardiac allograft vasculopathy was admitted for redo orthotopic heart transplantation (day 0).

The recipient's immediate post-transplant course was notable for cardiogenic shock, hemorrhage prompting prolonged open chest, and acute kidney injury requiring renal replacement therapy. He was placed on antimicrobial prophylaxis with sulfamethoxazole-trimethoprim, inhaled amphotericin B, and acyclovir in accordance with institutional protocols. After chest closure on day 5, the recipient was noted to be mildly confused and had a persistent leukocytosis without focal complaints.

#### 2.1.2 Donor history

The organ donor was a man in his 30s with a history of intravenous drug use, who was found unconscious with a package of white crystalline substance thought to be methamphetamines adjacent to his body. During his hospitalization at another facility, he progressed to brain death in the setting of intracranial hemorrhage. Organ procurement was performed three days after the donor's admission. The heart organ was maintained on a TransMedics device.

#### 2.1.3 Physical examination, laboratory findings, and radiology results

At time of Infectious Diseases consultation on day 6, the recipient was afebrile and hemodynamically stable. His exam was notable for mild lethargy, clean and intact central line insertion sites, serosanguinous chest tube drainage, absence of erythema or induration surrounding incision sites, and mild bilateral pedal edema. Laboratory studies included the following: white blood cell count of 28,100 cells/microL, hemoglobin of 9.5 g/dL (stable post-operatively), platelet count of 75/microL, and creatinine of 2.2 ml/dL on continuous renal replacement therapy.

**Fig. 1:**
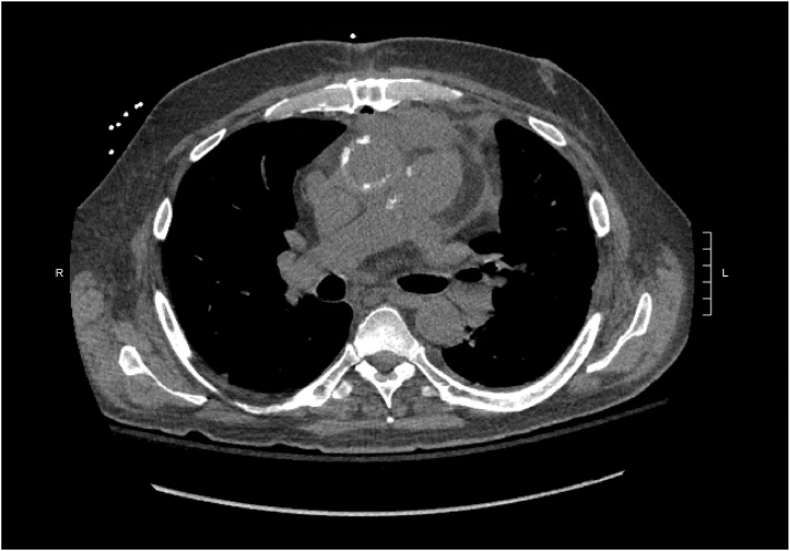
CT scan of the chest demonstrating a six-centimeter retrosternal fluid and gas collection in the operative fie.

Post-operative transesophageal echocardiography demonstrated normal left ventricular function, moderate right ventricular dysfunction, and mild mitral regurgitation without evidence of vegetations or other valvular abnormalities. Chest radiography was unremarkable. A CT of the chest (Fig. 1) was obtained and demonstrated a six-centimeter retrosternal fluid and gas collection in the operative field.

Donor blood and renal tissue cultures obtained at the outside facility were reviewed and notable for growth of *Candida parapsilosis* complex. Concurrently, *Candida parapsilosis* complex was identified in recipient blood cultures at our institution via matrix-assisted laser desorption/ionization time-of-flight (MALDI-TOF).

#### 2.1.4 Treatment

The patient was started on micafungin 100 mg daily and underwent removal of all lines. Repeat transthoracic and later transesophageal echocardiography showed no evidence of valvular vegetations or abnormalities of any sites of anastomosis. Ophthalmologic examination showed no evidence of fungal endophthalmitis.

The patient remained persistently fungemic with *Candida parapsilosis* complex after 7 days despite pursuing a line holiday and increasing micafungin dosing to 150mg daily. Despite five sternal wound washouts, another line holiday, and ongoing micafungin therapy over the upcoming weeks, the recipient's blood and tissue cultures through post-operative day 25 continued to grow *Candida parapsilosis* complex. Liposomal amphotericin B 3 mg/kg daily was added to micafungin 150mg daily, and blood cultures cleared by post-operative day 30.

Susceptibilities for the *Candida parapsilosis* complex returned with the following minimum inhibitory concentrations (MICs): fluconazole 32 mcg/mL (resistant by Clinical and Laboratory Standards Institute guidelines [CLSI]), micafungin 0.25 mcg/mL (susceptible), and voriconazole 0.016 mcg/mL (susceptible). No CLSI breakpoints were available for amphotericin B. The liposomal amphotericin B was discontinued approximately four weeks after blood culture clearance, and the recipient was maintained on micafungin 100 mg daily for close to 6 weeks after discharge with persistent culture negativity thereafter.

#### 2.1.5 Outcome

As an outpatient, the recipient was started on voriconazole and bridged with micafungin for close to 3 weeks. His voriconazole levels initially ranged from 1.1 to 1.9 mcg/mL for two to three weeks but subsequently settled between 2 and 3 mcg/mL.

Two months after transitioning to voriconazole and nearly five months after his last positive blood culture, the recipient reported a two-week history of fatigue and diffuse myalgias to his Infectious Diseases provider. Blood cultures were repeated and returned positive for *Candida parapsilosis* complex. He was readmitted, and micafungin 150 mg daily was added to voriconazole. The patient underwent an extensive work-up for persistent source of infection including repeat transthoracic and transesophageal echocardiography, venous duplex studies, and positron emission tomography scan, all of which did not reveal an obvious endovascular source of infection. Because of mild non-specific FDG-avidity of his post-operative sternal fragments, the patient underwent a sternal bone biopsy which did not show pathologic or microbiologic evidence of fungal organisms.

The recipient was transitioned from voriconazole and micafungin to liposomal amphotericin B 3mg/kg daily and micafungin. Flucytosine 25 mg/kg four times daily was added after persistent culture positivity at two weeks of therapy. Repeat voriconazole MIC testing revealed an increase to 2 mcg/mL from 0.016 mcg/mL, concerning for the development of voriconazole resistance. Ultimately, his fungemia cleared on liposomal amphotericin B, micafungin, and flucytosine after nearly 3 weeks of positive blood cultures. He was transitioned to posaconazole, micafungin, and flucytosine on discharge for four more weeks, and he was ultimately maintained on posaconazole and flucytosine to this day without relapse.

### 2.2 Donor-derived *Aspergillus* infection

#### 2.2.1 Patient presentation

A 59-year-old man was admitted for orthotopic heart transplantation (day 0). His past medical history was notable for non-ischemic cardiomyopathy status post implantable cardiac defibrillator (ICD) and prior sternotomy for implantation of a left ventricular assist device (LVAD).

During the transplant surgery, the organ recipient's ICD and LVAD were removed successfully. The donor heart was inspected with normal appearance of the aortic, mitral, and tricuspid valves. Post-operatively, the transplant recipient received empiric vancomycin and piperacillin-tazobactam along with routine antimicrobial prophylaxis based on institutional post-transplantation protocols. On day 2, the transplant surgery team was notified that the gross pathology from donor kidneys and adrenal glands returned with micro-abscesses.

#### 2.2.2 Donor history

One month prior to his death, the male organ donor in his 30's presented to his primary care physician with weight loss and decompensated cirrhosis. He had a profound leukocytosis to 40,000 cells/microliter at that time but did not undergo further evaluation. Subsequently, he suffered a motor vehicle accident, was admitted to a different institution, and progressed to brain death due to multifocal intracranial hemorrhage, which had a radiographic distribution that could have been consistent with an embolic phenomenon. At the time of hospital presentation after the motor vehicle accident, his profound leukocytosis was still present (40,900 cells/microliter). Pre-transplantation work-up of the donor included routine blood cultures and echocardiography, which were unremarkable.

#### 2.2.3 Physical examination, laboratory findings, and radiology results

At time of Infectious Diseases consultation on day 3, the recipient was febrile to 38.4 Celsius over the past 24 hours and tachycardic to 100/min but otherwise hemodynamically stable on minimal ventilator settings. Exam was notable for clean and intact central line sites, serosanguinous chest tube drainage, absence of erythema or induration surrounding incision sites, and minimal pitting edema. Laboratory findings were notable for the following: white blood cell count of 19,100 cells/microliter; hemoglobin of 10.2 g/dL (stable post-operatively); platelet count of 232/mL; creatinine 1.4 (baseline 0.9) mg/dL; AST of 126 U/L; ALT of 33 U/L; Alkaline Phosphatase 38 U/L; and Total Bilirubin 2.5 mg/dL. Post-operative transesophageal echocardiography (TEE) on day 2 demonstrated normal left ventricular function without valvular abnormalities of note.

Further preliminary histopathology review of the donor's left and right kidneys demonstrated hyaline hyphal forms. The recipient was started on micafungin 150 mg intravenous (IV) daily by day 3, pending further details from the donor's histopathology and cultures. Overall, the recipient was clinically improving without evidence of invasive fungal infection clinically or radiographically.

Cultures from donor kidney and adrenal samples in addition to bronchoalveolar lavage cultures at the outside hospital confirmed *Aspergillus fumigatus* on day 4. Methods used to identify *A. fumigatus* at the outside hospital were not specified in the microbiology reports.

#### 2.2.4 Treatment

Isavuconazole therapy (372 mg every 8 hours for six doses followed by 372 mg daily) was initiated in the recipient as a precautionary measure on day 4, immediately after learning of the histopathology results. Micafungin therapy was discontinued shortly thereafter. The recipient was extubated and weaned from inotropes on post-operative day 4; his incision site after surgical bandage removal on post-operative day 6 appeared healthy. He had no further fevers with a stable leukocytosis around 13,000/mL.

**Fig. 2:**
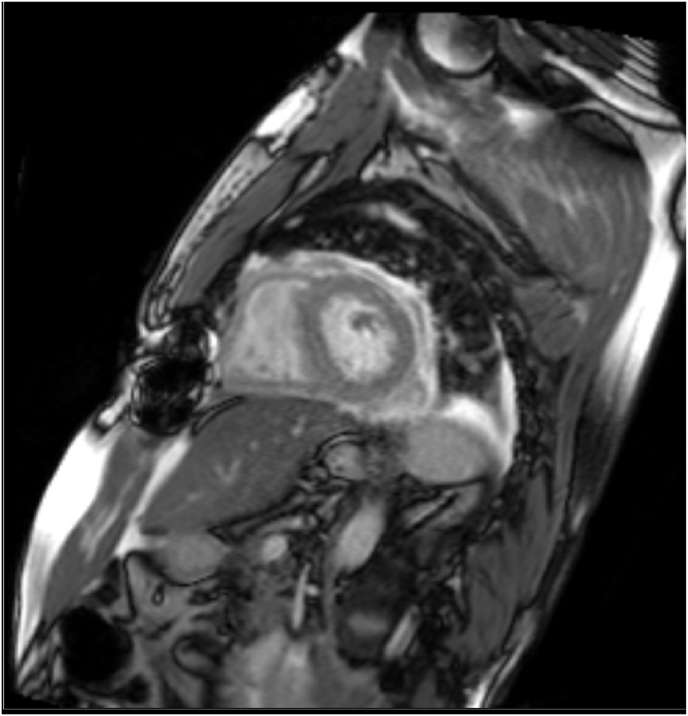
Cardiac MRI with small LV thrombus adhered to mid-to-distal inferior wall measuring 1.1x1.0x0.8cm.

On post-operative day 6, the recipient developed mild hypoactive delirium without focal neurologic deficits, followed by a mounting leukocytosis. A non-contrasted head CT scan on day 6 demonstrated a small acute left corpus striatum infarct, and subsequent brain MRI on post-operative day 9 showed numerous acute infarcts throughout the bilateral hemispheres concerning for embolic phenomena. A post-operative cardiac MRI obtained concurrently demonstrated a left ventricular thrombus along the inferior septal wall though ventricular and valvular morphology and function were unremarkable (Fig. 2).

Transesophageal echocardiogram on day 9 showed normal valvular morphology and confirmed a mobile mass on the mid-distal inferoseptal wall consistent with thrombus. Lastly, a CT scan of the chest, abdomen, and pelvis on day 10 demonstrated a small partially loculated pericardial effusion, a newly developed retrosternal fluid collection, and bilateral renal wedge-shaped hypodensities concerning for multifocal infarction (Fig. 3a, Fig. 3ba and b).

**Fig. 3a:**
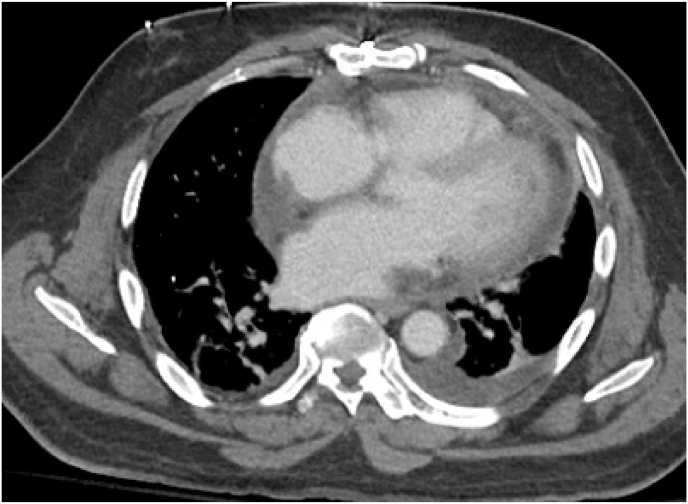
CT image of the thorax with loculated pericardial effusion.

**Fig. 3b:**
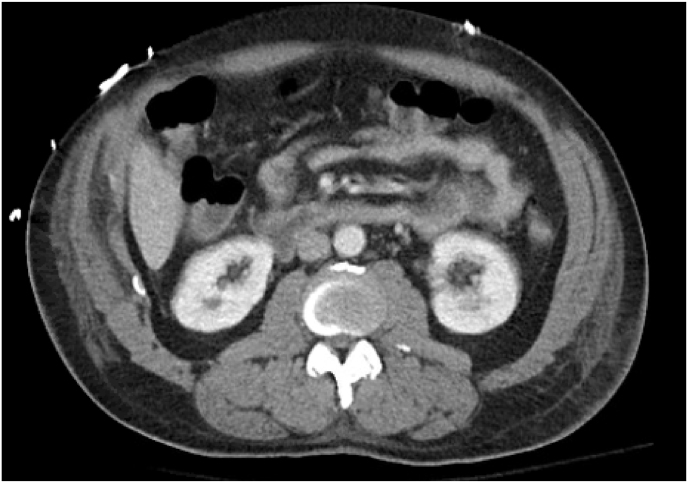
CT image of the abdomen with bilateral wedge-shaped renal infarcts.

Serum galactomannan antigen testing returned low-positive at 0.568 index units (IU) (Platelia enzyme immunoassay; cutoff <0.5 IU) on day 9. Fungal blood cultures and later mediastinal tissue cultures from the recipient grew *Aspergillus fumigatus* as identified by MALDI-TOF. The fungal blood culture isolate was sent for MIC testing with the following results: voriconazole 0.25 mcg/mL (susceptible per CLSI M38), posaconazole 0.06 mcg/mL (no established breakpoints), isavuconazole 0.5 mcg/mL (no established breakpoints), micafungin ≤0.015mcg/mL (no established breakpoints), and amphotericin B 0.5 mcg/mL (no established breakpoints).

On day 9, the isavuconazole therapy was discontinued, and the patient was initiated on voriconazole 6mg/kg every 12 h for two doses followed by 4 mg/kg every 12 h. Micafungin 150 mg IV daily was resumed.

#### 2.2.5 Outcome

The patient's mental status improved. However, after five days on the revised regimen (day 14), he developed a new right gaze preference and left-sided hemiparesis. CT imaging on day 14 revealed a large right intraparenchymal hemorrhage complicated by partial herniation requiring neurosurgical evacuation.

Starting on day 19, he underwent seven sternal debridement procedures to obtain mediastinal source control. Mediastinal tissue cultures collected with each debridement over 7 days grew *Aspergillus fumigatus*, but by the eighth day, these tissue cultures returned negative, and his chest was closed. He continued on voriconazole and micafungin for approximately 6 weeks, at which time he developed a significant liver injury that prompted a switch to posaconazole and micafungin.

Over his subsequent 11-month hospitalization, his course was complicated by line-associated bloodstream infections, *C. difficile* infection, acute cholecystitis, seizures, and COVID-19 infection. Nonetheless, his neurologic functional status slowly improved, with residual verbal communication difficulties and persistent partial left-sided hemiparesis.

## 3 Discussion

Here we describe two donor-derived invasive fungal infections among heart transplant recipients, including a rare occurrence of *Aspergillus fumigatus* transmission. In both cases, the organ donors presumably had undetected fungal endovascular infections prior to transplant, given evidence of metastatic infection including bilateral renal abscesses and, in the *Aspergillus* case, cerebral infarcts in a distribution suggestive of an embolic phenomenon. These endovascular infections were transmitted to the recipients, who both also developed fungemia, mediastinitis, and - in the *Aspergillus* case - a likely infected cardiac thrombus. A combination of prolonged antifungal therapy and surgical debridement were required to clear blood and/or tissue cultures in both cases with substantial residual morbidity.

### *3.1* *Candida parapsilosis* complex donor-derived infection

As the organ donor pool expands to include those with higher-risk comorbidities, infectious diseases providers and transplant surgeons must be vigilant for disseminated infections in the organ donor. Risk factors for invasive *Candida* infections include intravenous drug abuse, recent chemotherapy for cancer treatment, prolonged presence of central venous catheters, and prior valvular damage or surgery [5].

Management of *Candida* endocarditis, regardless of transplantation status, involves both surgical intervention with valve replacement and antifungal therapy with either high-dose echinocandin therapy or liposomal amphotericin B 3–5 mg/kg daily with or without flucytosine 25 mg/kg four times daily [[5], [6], [7]]. *Candida* mediastinitis should be managed similarly with both antifungal and surgical therapy including mediastinal debridement. However, even with appropriate therapy, mortality from *Candida* infective endocarditis is estimated to be between 25 and 50 % at one year with worse outcomes noted among those who do not undergo surgery [6,[8], [9], [10]].

The *Candida* case had two notable therapeutic complications: 1) persistent candidemia despite mediastinal debridement and antifungal monotherapy and 2) relapsed candidemia several months after culture clearance. While endovascular involvement was suspected during both presentations, no radiographic evidence of intracardiac or intravascular infection was found that could have been surgically targeted. Relapse of infection can occur with *Candida* endovascular infections, which in part may be due to the ability of *Candida* to form biofilms [8,9].

To limit these complications, combination antifungal therapy has been suggested for therapy of primary, refractory, and relapsed *Candida* endovascular infections for several reasons. These reasons include the potential for additive or synergistic efficacy, reduced risk of resistance, and ability to dose-reduce antifungals to limit toxicities [11]. While randomized trials have not been conducted, *in vitro* studies and case reports have suggested success with combination therapy. Liposomal amphotericin B, for instance, has shown promising *in vitro* synergistic activity with both echinocandins and posaconazole, and flucytosine is frequently employed in severe cases of invasive candidiasis (such as *Candida* meningitis) in combination with liposomal amphotericin B [10,11]. A combination azole-flucytosine step-down regimen was employed after our patient's infection relapsed.

### *3.2* *Aspergillus fumigatus* donor-derived infection

Donor-specific risk factors known to be associated with transmission of filamentous IFI to organ recipients include liver dysfunction, pre-existing immunocompromised state, prolonged ICU stay, prolonged mechanical ventilation, near-drowning experiences, and transplant tourism [3,12,13]. Notably, cirrhosis (independent of transplant status) is a significant risk factor for invasive aspergillosis, with estimated rates of IFIs due to *Aspergillus* species approaching 2.8 % among patients with cirrhosis [14]. These donor risk factors should be carefully considered in the assessment of transplant recipients, particularly if the donor has unexplained clinical findings such as pulmonary nodules, leukocytosis, or central nervous system lesions.

Management of *Aspergillus fumigatus* endocarditis in both transplant and non-transplant populations includes antifungal therapy as well as surgical interventions with valve replacement [15,16]; *Aspergillus* mediastinitis similarly requires both antifungal therapy and surgical debridement. Combination antifungal therapy may be employed, with voriconazole and/or liposomal amphotericin B as primary agents and echinocandins as adjunctive agents [15,17]. Embolization is a common complication of *Aspergillus* endocarditis that is estimated to occur in 45–75 % of affected patients, and mortality has been estimated to reach over 90 %, with highest risk of mortality occurring early after diagnosis and among those managed with medical therapy alone [15–18]. While aggressive mediastinal debridement was pursued for source control with our patient, the presence of intra-cardiac thrombus and absence of valvular abnormalities did not offer clear nor safe surgical targets for further intervention.

### 3.3 Conclusions

In summary, donor-derived IFIs among heart transplant recipients are rare events with often devastating consequences. The donor's medical history should be evaluated for unexplained clinical findings, and any evidence of fungal infection in the donor warrants meticulous evaluation for IFI in the recipient. Organ recipients should be managed accordingly, which in the heart transplant recipient necessitates evaluation for and management of fungal endocarditis. Both antifungal therapy and surgical interventions tailored to the affected organ are frequently required for therapy of donor-derived IFI, but prognosis may be grim. Prevention of transplant-related IFI is a necessary area for further study including microbiologic testing of organ tissue and preservation fluid via culture or rapid molecular testing.

## CRediT authorship contribution statement

**Jay Krishnan:** Writing – original draft, Visualization, Data curation, Conceptualization. **Manuela Carugati:** Writing – review & editing. **Rachel A. Miller:** Writing – review & editing. **Cameron R. Wolfe:** Writing – review & editing. **John R. Perfect:** Writing – review & editing. **Julia A. Messina:** Writing – original draft, Visualization, Project administration, Data curation, Conceptualization.

## Declaration of competing interest

The authors have no competing interests related to this work to declare.
